# ERK2, but Not ERK1, Mediates Acquired and “*De novo*” Resistance to Imatinib Mesylate: Implication for CML Therapy

**DOI:** 10.1371/journal.pone.0006124

**Published:** 2009-07-01

**Authors:** Clara I. Aceves-Luquero, Anupriya Agarwal, Juan L. Callejas-Valera, Laura Arias-González, Azucena Esparís-Ogando, Luis del Peso Ovalle, Itxaso Bellón-Echeverria, Miguel A. de la Cruz-Morcillo, Eva M. Galán Moya, Inmaculada Moreno Gimeno, Juan C. Gómez, Michael W. Deininger, Atanasio Pandiella, Ricardo Sánchez Prieto

**Affiliations:** 1 CRIB/Facultad de Medicina, UCLM, Albacete, Spain; 2 Division of Hematology and Medical Oncology, Oregon Health and Science University Cancer Institute, Portland, Oregon, United States of America; 3 Instituto de Biología Molecular y Celular del Cáncer, CSIC-USAL, Salamanca, Spain; 4 Departamento de Bioquímica, Facultad de Medicina, Universidad Autónoma de Madrid, Madrid, Spain; Roswell Park Cancer Institute, United States of America

## Abstract

Resistance to Imatinib Mesylate (IM) is a major problem in Chronic Myelogenous Leukaemia management. Most of the studies about resistance have focused on point mutations on *BCR/ABL*. However, other types of resistance that do not imply mutations in *BCR/ABL* have been also described. In the present report we aim to study the role of several MAPK in IM resistance not associate to *BCR/ABL* mutations. Therefore we used an experimental system of resistant cell lines generated by co-culturing with IM (K562, Lama 84) as well as primary material from resistant and responder patient without *BCR/ABL* mutations. Here we demonstrate that Erk5 and p38MAPK signaling pathways are not implicated in the acquired resistance phenotype. However, Erk2, but not Erk1, is critical for the acquired resistance to IM. In fact, Bcr/Abl activates preferentially Erk2 in transient transfection in a dose dependent fashion through the c-Abl part of the chimeric protein. Finally, we present evidences demonstrating how constitutive activation of Erk2 is a *de novo* mechanism of resistance to IM. In summary our data support the use of therapeutic approaches based on Erk2 inhibition, which could be added to the therapeutic armamentarium to fight CML, especially when IM resistance develops secondary to Erk2 activation.

## Introduction

Chronic Myeloid Leukaemia (CML) is one of the most studied human malignancies. The disease is originated in a hematopoietic stem cell progenitor by a reciprocal translocation between chromosome 9 and 22 t(9∶22)(q34∶11) joining the *c-ABL* tyrosine kinase on chromosome 9, and the Break point Cluster Region (*BCR*) on chromosome 22. This generates a chimeric protein with deregulated activity, which plays an essential role in the pathogenesis of the disease (for review see [1]).

Deregulation induced by Bcr/Abl protein affects the function of several signaling pathways implicated in the malignant phenotype. Consistent with this, alterations in most of the members of the serine-threonine kinases Mitogen Activated Protein Kinases (MAPKs) has been demonstrated, explaining several properties associated to *BCR/ABL* transformation, including survival or drug resistance [2]–[4]. A major breakthrough in CML therapy has been the design and development of specific inhibitors for Bcr/Abl tyrosine kinase activity. The first one, Imatinib Mesylate (IM, Gleevec™, formerly known as STI-571), has been widely studied (for review[5]). This potent and specific chemical inhibitor interacts with the ATP binding pocket of c-Abl, blocking the tyrosine kinase activity associated with the chimeric protein. However, in spite of the successful clinical results obtained with IM, a major problem is the generation of resistance. Some resistant phenotypes to IM have been explained by point mutations in several domains of the chimeric protein, especially those that affect the tyrosine kinase domain [6]. This has prompted the generation of new therapeutic agents to overcome this type of resistance [7], [8]. Futhermore, IM has also been reported to fail to control disease progression in the absence of *BCR/ABL* mutations, an aspect that remains poorly understood [9], [10]. Several mechanisms, such as the overexpression of Bcr/Abl, increases in drug efflux transport proteins or the overexpression of other members of the Src tyrosine kinase family have been proposed to explain the failure to respond to IM (for a review [11]). Drug resistance can be divided in two general types: *de novo*, presents in patients who are resistant to therapy from the beginning of the treatment; and acquired resistance, that occurs in those that develop resistance during treatment. In this sense, continuous exposure to IM allowed us to obtain an experimental system that mimics the acquired resistance ([12]–[14]). Interestingly, *de novo* and acquired resistance to IM in CML seem to have a convergence point in the Bcr/Abl overexpression, which is a common feature of the two last phases of the disease [15]–[17].

In this scenario, we decided to evaluate the role of several members of the MAPKs superfamily in the acquired resistance to IM using the above mentioned experimental system. Our results demonstrate a critical role for Extracellular Signal-Regulated Kinase (ERK) 2 in the acquired resistance to IM, excluding other MAPK such as p38 and Erk5. The proposed role for Erk2 not only applies to acquired resistance, our data demonstrate that constitutive activation of this particular MAPK can render *de nov*o resistance. These results could also explain the clinical failure observed in the advanced stages of the CML in which the high Bcr/Abl expression level is a critical event.

## Materials and Methods

### Ethics Statement

The Investigation committee of the Universidad de Castilla La Mancha approved the present research. All patients analyzed in this study were treated at Oregon Health & Science University and have given written informed consent for the study according to the Declaration of Helsinki.

### Chemicals and antibodies

Antibodies against the phosphorylated forms of p38MAPK, Erk5 and Erk1/2 were purchased from Cell Signaling Technology. Antibodies against the non-phosphorylated forms of Bcr, p38, Erk1 (K-23) or Erk2 (C-14) were purchased from Santa Cruz Biotechnology. Antibodies against the non phosphorylated form of Erk5 were purchased from Cell Signaling Technology, or obtained in our laboratory [18]. HA antibody was purchased from Babco. Antibody against c-Abl was from BD Biosciences. Phosphotyrosine (4G10) was from Upstate Biotech. PD98059 and SB203580 were from Calbiochem. BIRB0796 was obtained from the University of Dundee. IM was kindly supplied by Elisabeth Buchdunger (Novartis-Pharma, Basel, Switzerland).

### Cell Lines and Plasmids

293 T cells were maintained in 5% CO_2_, at 37°C in DMEM (Biowhitaker) supplemented with 10% FBS plus antibiotics (Biowhitaker). Lama84, K562 cells and resistant derivates (a generous gift from Dr. J. V. Melo, Institute of Medical & Veterinary Science Adelaide, Australia) were maintained in RPMI supplemented with 10% FBS plus antibiotics. Resistant cells to IM were routinely maintained in the presence of 1 µM of this inhibitor. ·32D cells harboring *K-Ras* wt and mutant forms have been previously described [19]. The plasmid for constitutively active HA tagged *MEK1* was kindly supplied by Dr. R. Pulido (CIPF, Valencia, Spain). The expression vector for *BCR/ABL* was kindly supplied by Dr J. Pear (USF, California, USA). Plasmids for Green Fluorescent Protein *(GFP)-ABL* and *BCR* have been previously described [3], [20].The plasmid for GFP-tagged *PEA15* was a generous gift from Dr. H Chneiweiss (Institut National de la Sante et de la Recherche Medicale, Paris, France). shRNA for *ERK5* and luciferase in pRetro-QSiren was kindly provided by Dr. M. Villalba (IGMM, CNRS, Montpellier, France). ShRNA for *ERK2* and *ERK1* in PLKO-PURO vector were purchased from Sigma. We selected the best performing clone shRNA for further analysis, as judged for functional interference with exogenous tagged protein by western blotting.

### Patient samples

Mononuclear cells (MNCs) were isolated from patient bone marrow aspirates or peripheral blood samples by Ficoll density gradient centrifugation. For direct immunoblots, 2 million cells were lysed in sample buffer (75 mM Tris pH 6.8, 3% SDS, 15% glycerol, 8% β-mercaptoethanol, 0.1% bromophenol blue) and whole cell lysates were separated by SDS-PAGE. Patient samples utilized in this study are wild type for *BCR-ABL* covering the Cap, SH3, SH2 and kinase domains of *ABL* (exons 1–9), *KRAS* (exons 3–5), *NRAS* (exons 2–5), *PTPN11* (exons 3, 4, 8 and 13), *KIT* (entire coding region), *FLT3* (exons 14 and 20), *JAK2* (exon 14), *PDGFRα* (exons 12, 14 and 18) and *PDGFRβ* (exons 11 and 17

### Western blotting and immunoprecipitation assays

For ERK1/2 and p38MAPK detection, cells were collected in lysis buffer containing 25 mM HEPES, pH 7.5, 0.3 M NaCl, 1.5 mM MgCl_2_, 0.2 mM EDTA, 1% Triton X-100, 0.1% SDS, 0.5% Deoxycholic Acid, 20 mM β-glycerolphosphate. For ERK5 detection, cells were collected in 100 mM HEPES, pH 7.5, 50 mM NaCl, 0,1% Triton X-100, 5 mM EDTA, 0,125M EGTA. In both cases protease and phosphatase inhibitors (0.2 µg/ml Leupeptin,2 µg/ml Aprotinin, 1 mM PMSF and 0.1 mM Na_3_VO_4_) were added. The indicated amounts of protein were loaded onto 10% SDS-PAGE, transferred to nitrocellulose filters and blotted against different proteins using specific antibodies against the phosphorylated form or the total protein. In the immunoprecipitation assays, extracts were precleared and soluble fractions were incubated with the indicated antibody. After 2 hours, extracts were incubated for 45 minutes in the presence of protein G (Gamma bind Sepharose, Pharmacia Biotech) and then washed 3 times in the same lysis buffer. Then immunocomplexes were resuspended in loading buffer, and loaded onto SDS-PAGE gels. Images show a representative blot out of three experiments with nearly identical results.

### Transfections

293 T were transiently transfected by using Lipofectamine (Invitrogen) following manufacturer's instructions. The total amount of DNA was normalized using an empty vector. Cells were lysed 36 h after transfection and samples were processed for immunoprecipitation or Western blot as previously described. In the case of stably transfected, K562 cells, we used the same protocol with Lipofectamine. 36 h after transfection positives clones were selected with Puromycin (5 µg/ml) or G418 (650 µg/ml) both purchased from Sigma.

### Proliferation assays

Proliferation was evaluated by MTS method using CellTiter 96 AQ Non-Radiactive Cell Proliferation Assay (Promega) following manufacturer's instructions. The colorimetric reagent was added to each well, incubated for 1–4 h at 37°C in 5%CO2 and the absorbance values read at 490 nm. Statistical significance was obtained by using an unpaired t test running in Graph PAD prism program.

### Immucytochemistry

K562 cells were plated onto sterile coverslips treated with polilysine (Sigma-Aldrich). Then samples were fixed in paraformaldehide (4%) at 4°C for 5 minutes and incubated in PBS buffer containing 10% normal goat serum and 0.3% Triton X-100 (blocking buffer). Samples were then incubated with pErk1/2 overnight and, after extensive wash; incubated 45 minutes with Alexa fluor 546 conjugated anti-mouse antibodies (Molecular Probes) counterstained with DAPI (Sigma) and mounted with Fluorosave (Calbiochem). Positive immunoflorescence was detected in a Leica-DMRXA-photomicroscope.

## Results

### IM resistant cell lines display higher levels of Bcr/Abl than parental cells, but their tyrosine kinase activity is blocked by IM

**Figure 1:**
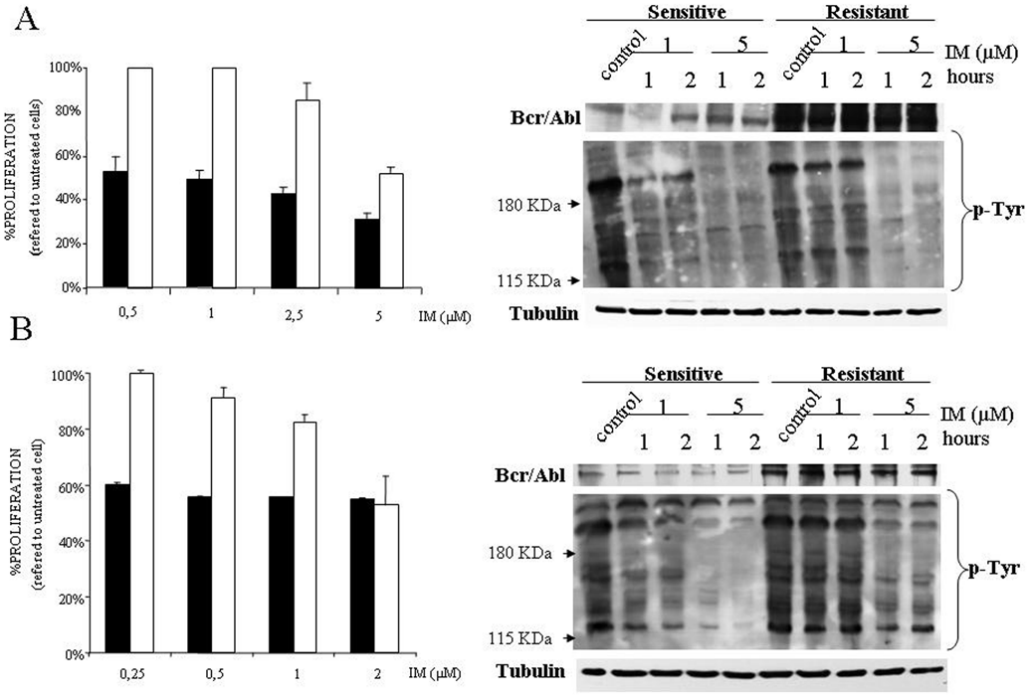
IM resistant cell lines display higher levels of Bcr/Abl than parental cells. (A) Sensitive (black bars) and resistant (white bars) K562 cell lines were exposed to several concentrations of IM for 48 hours; after which viability was determined by MTS assays. The histogram represents at least 3 independent experiments performed in triplicate cultures. Bars: mean±Standard Deviation. Right panel, K562 sensitive and resistant cell lines were treated with IM 1 or 5 µM for 1 and 2 hours, then lysed and blotted against the indicated primary antibodies by using 100 µg of total cell lysates. As a loading control membranes were re-probed against tubulin. (B) Same as in A for Lama84 cell lines.

To analyze the role of several members of the MAPK family in the acquired resistance to IM we used two cell lines, K562 and Lama84, and their resistant derivates generated by co-culturing with IM [13]. First, we evaluated the action of IM on the viability of sensitive and resistant cell lines. IM had a significantly higher action on sensitive K562 (fig. 1A) and Lama84 cells (fig. 1B). Next, we evaluated Bcr/Abl expression levels, that were higher in resistant cells (fig. 1A and B), according to previous reports [12]. We evaluated the inhibition of tyrosine phosphorylation by IM in resistant and sensitive cell lines (fig. 1A and B). As expected resistant cell lines, considering that cells growth in the presence of 1 µM, showed no effect by the incubation with this dose of IM, while all the cell lines tested showed a marked decrease in the overall Tyr-phosphorylation pattern after a short exposure to IM 5 µM. Indeed, the same results were obtained in an experimental model of murine Baf3 cells, expressing an exogenous *BCR/ABL*, in which resistant cells displayed higher levels of Bcr/Abl that is inhibited by IM (data not shown). This set of experiments indicates that in our experimental system resistance correlates with an increase in Bcr/Abl expression level and also excludes mutations in the ATP binding site.

### Basal activation of Erk1/2 correlates with resistance to IM

**Figure 2:**
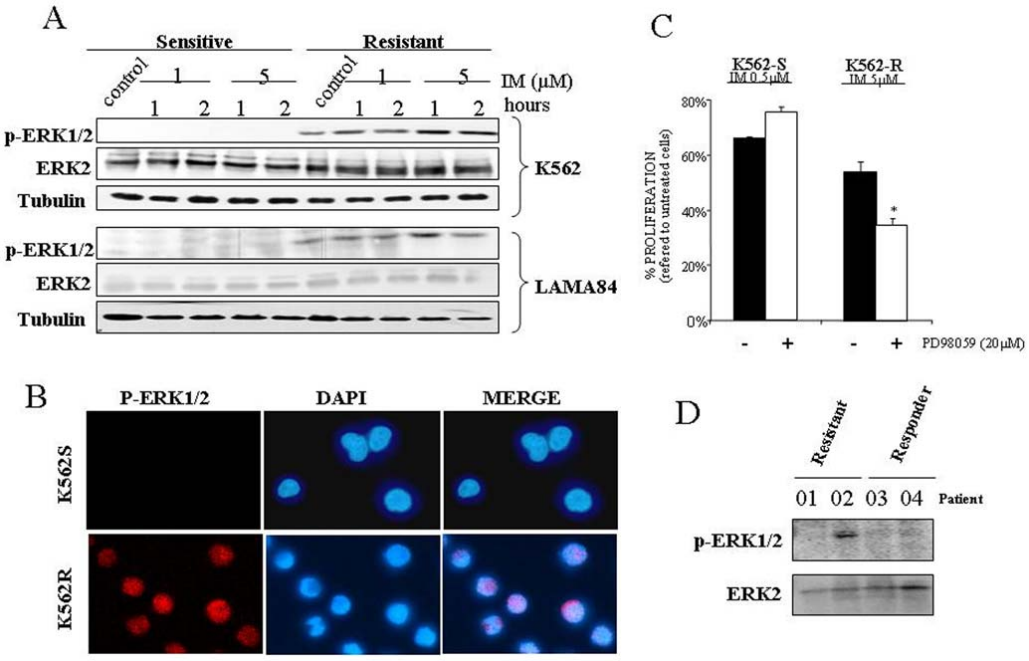
Basal activation of Erk1/2 correlates with resistance to Imatinib. (A) Sensitive and resistant K562 and Lama84 cell lines were treated with the indicated concentrations of IM for 1 and 2 hours, lysed and analyzed by immunoblotting against active and total Erk1/2 by using 50 µg of total cell lysates. As a loading control membranes were re-probed against tubulin. (B) Immunofluorescence detection of active Erk1/2 in K562. The image shows the most representative experiment out of three performed with nearly identical results. (C) K562 cells, sensitive (K562-S) and resistant (K562-R), were incubated with IM 0.5 (for sensitive) or 5 µM (for resistant) in the presence (white bars) or absence (black bars) of the indicated amounts of PD98059. Viability was referred to controls treated only with PD98059 (in the case of co- incubation with PD) or vehicle (DMSO) in the case of IM. Treatment with PD98059 alone induces toxicity lower that 3% in resistant and sensitive cell line. The histogram represents at least 3 independent experiments performed in triplicate cultures. Bars: mean±Standard Deviation. (D) MNCs from CML patients primary resistant to IM (01, 02) and responder (03. 04) were analyzed by immunoblotting against active Erk1/2. Membrane was re-probed against total Erk2 as a loading control. Bars: mean±Standard Deviation. * p value of 0,045.

Erk1/2 have been related to IM acquired resistance [12]. Interestingly, hyperactivation of Erk1/2 was detected in the resistant cells lines while remained almost inactive in the sensitive cell lines (fig. 2A). Immunofluorescence studies (fig. 2B), indicate that active Erk1/2 was present in the nucleus of resistant cells, but not in the IM sensitive cell counterpart. In fact, the nuclear localization of Erk1/2 is a critical issue for IM resistance (see Figure S1). Furthermore, the use of a specific inhibitor of the Erk1/2 signalling pathways, induces an increase of the toxicity associated to IM only in the resistant cells (fig. 2C), corroborating the critical role of Erk1/2 in response to IM. In fact, similar results were obtained in the Lama84 experimental system (Figure S2). In an attempt to extrapolate our cell culture observation to human primary material, we also analyzed the activation status of Erk2 in few samples from IM resistant CML patients -with no mutation in *BCR/ABL*- and IM responders. Interestingly none of the responders -with low level of Bcr/Abl- had clear activation of Erk1/2 (fig. 2D and Figure S3). In contrast, and according to the data obtained in the cell lines, the only primary resistant patient with high Bcr/Abl levels (as determined by cytogenetic analyses) showed marked Erk1/2 phosphorylation (fig. 2D and Figure S3). Therefore, the data presented support the link between Erk1/2 activation, high Bcr/Abl expression level and IM resistance.

### Erk2 activation, but not Erk1, provokes acquired resistance to IM

**Figure 3:**
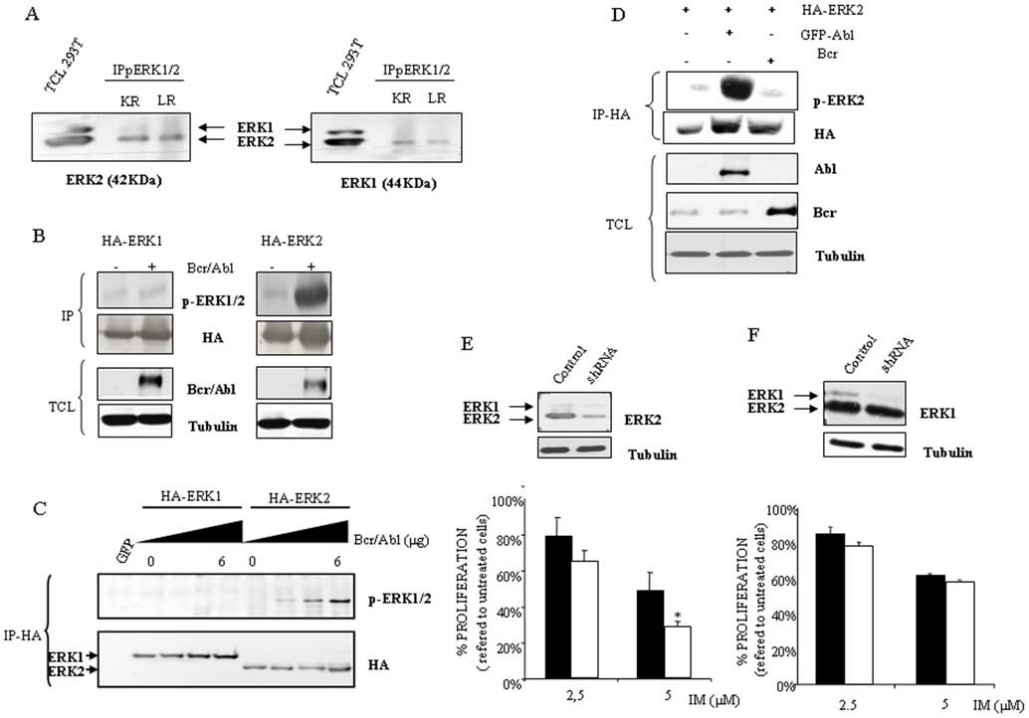
Erk2 activation mediates acquired resistance to IM. (A) Resistant K562 and Lama84 cell lines were lysed and immunoprecipitated against active forms of Erk1/2. Inmunocomplexes were blotted against Erk1 or Erk2 antibodies. Total cell lysated from 293 T cell line (50 µg) was used as a control for Erk1 or Erk2. KR and LR mean K562 resistant and Lama 84 resistant respectively. (B) 293 T cells were transiently co-transfected with *HA-ERK1* or *HA-ERK2* (0.5 µg), with or without *BCR/ABL* (4 µg) using lipofectamine. Next day cells were O/N starved. Then cells were collected in lysis buffer, inmunoprecipitated against HA and immunocomplexes were blotted against phospho-Erk1/2 and re-probed against HA. Bcr/Abl expression was evaluated by western blotting in total cell lysates. (C) 293 T cells were transiently transfected as in B with *HA-ERK1* or *HA-ERK2* (0.5 µg), plus *BCR/ABL* (0, 2, 4 or 6 µg) by using lipofectamine. As a control, cells were tranfected with a plasmid coding for *GFP*. 36 hours after transfection cells were collected in lysis buffer for Erk1/2 and immunoprecipitated against HA and blotted with phospho-Erk1/2 and HA. (D) 293 T cells were transiently co-transfected with *HA-ERK2* (0.5 µg), plus *ABL* or *BCR* (2 µg) using lipofectamine. 36 hours after transfection cells were collected in lysis buffer and immunoprecipated against HA. Immunocomplexes were blotted against phospho-Erk2 and re-probed against HA. Bcr and c-Abl expression was evaluated by Western blotting in total cell lysates. (E) K562 resistant cells were transfected with empty vector-PLKO-puro- (black bars) or shRNA against *ERK2* (white bars) were exposed to the indicated concentrations of IM, 48 hours later the viability was determined by MTS assays. The histogram represents at least 3 independent experiments performed in triplicate cultures. Bars: mean±Standard Deviation. Upper panels, Knock down of *ERK2* in K562 resistant cells through shRNA was evaluated by Western blotting. As a loading control membranes were re-probed against tubulin. (F) Same as in D but with *ERK1*shRNA. * p value of 0,045.

Interestingly, in our prior observation we detected only a single major band for Erk1/2 that was referred to as phospho-Erk1/2. To gain further insight into the characterization of the band detected by the anti-pErk1/2 antibody, we performed immunoprecipitation assays in resistant cell lines, using antibodies against active (phosphorylated) Erk1/2 and then immunocomplexes were blotted against both MAPKs, showing a pattern consistent with Erk2 as the use of total cell lysate from 293 T cells indicate (fig. 3A). This apparent specific activation of Erk2 could be initially explained by the different expression levels between both MAPKs. However, we performed transient transfection approaches to reach similar expression levels of Erk1 and Erk2. Interestingly, Bcr/Abl preferentially activated Erk2 with almost no effect on Erk1 (fig. 3B). Indeed, this effect was found to be dose dependent (fig. 3C). Furthermore, we performed transient transfection assays with *HA-ERK2* plus *BCR* or *c-ABL*, showing that Erk2 activation mediated by Bcr/Abl is due to the c-Abl part of the chimeric protein (fig. 3D).

Although this set of experiments indicated a marked preference of Bcr/Abl for Erk2 activation and a correlation with IM resistance, they did not allow us to conclude a direct cause-effect mechanism yet. Therefore, to demonstrate the role of this particular MAPK we used RNAi technology to knock down the expression of *ERK2*. K562 resistant cells were transfected with a plasmid coding for an *ERK2* shRNA and its control vector. Selected mass cultures were analyzed for Erk2 expression levels and viability in response to IM. As shown (fig. 3E), a clear decrease in Erk2 expression was achieved in cells transfected with *ERK2* shRNA, that rendered a reduction in the resistance to IM. Using an analogous experimental setup, we also explored whether Erk1 could have a role in IM resistance. Cells were transfected with shRNA to knock down *ERK1* expression, and the selected cultures were analyzed for expression levels and response to IM (fig. 3F). In these experiments, knock down of *ERK1* did not significantly affected IM action, supporting the critical role for *ERK2*.

In summary, the presented experiments clearly support that acquired resistance to IM observed is due to, at least in part, the hyper activation of the Erk2 signaling pathway that correlates with the high levels of Bcr/Abl.

### Erk2 activation mediates “de novo” resistance to IM

**Figure 4:**
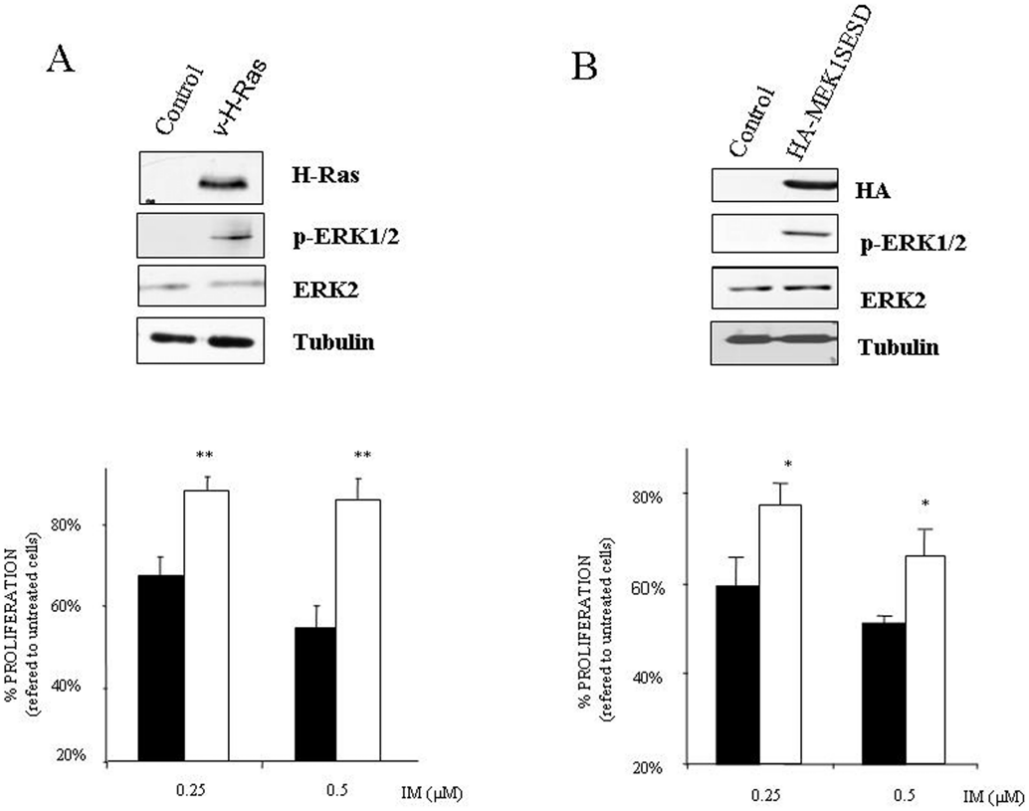
Erk2 mediates *de novo* resistance to IM. (A) Sensitive K562 cells were retrovirally transduced with a mutant form of *H-Ras*. Selected cultures were analyzed in terms of v-H-Ras expression and Erk2 activation. As a loading control tubulin was evaluated. K562 control (black bars) and v-H-Ras expressing cells (white bars) were exposed to the indicated concentrations of IM. 48 hours after this treatment the percentage of survival cells was determined by MTS assay. The histogram represents at least 3 independent experiments performed in triplicate cultures. Bars: mean±Standard Deviation. (B) Sensitive K562 cells were stably transfected with empty vector PCDNA3 (control) or PCDNA3 HA-tagged constitutively active *MEK1*. Expression and functionality was tested by western blotting against HA and active Erk2 respectively. As a loading control tubulin was evaluated. K562 control (black bars), and HA-Mek1 hyperactive cells (white bars) were exposed to the indicated concentrations of IM. 48 hours after this treatment the percentage of survival cells was determined by MTS assay. The histogram represents at least 3 independent experiments performed in triplicate cultures. Bars: mean±Standard Deviation. ** p value<0,007, *p value<0.02.

In light of our findings we next asked if Erk2 activation could induce *de novo* resistance. First, we used a mutated form of *H-Ras* (v-H-Ras), probably one of the best natural activators of Erk1/2 signaling pathway [21], that was retrovirally transduced onto sensitive K562 cells. Selected pools were evaluated in terms of v-H-Ras expression, Erk1/2 activation and IM resistance. As it is shown (fig. 4A), expression of v-H-Ras induced a marked increase in Erk2 activation that correlates with IM resistance. In fact, the same results have been obtained by using mutant forms of *K-Ras*, known to induces resistance to IM [19], in 32Dp210^BCR-ABL^ cells as well as in a CML patient sample with a weakly activating T58I *K-Ras* mutation, (Figure S4). However, overexpression of mutant Ras proteins can affect other signalling pathways such as PI3K/Akt [22], that has been related to IM resistance [23], [24]. Therefore, to exclusively activate Erk2 without affecting other signaling pathways, K562 sensitive cells were stably transfected with an exogenous constitutively active *MEK1*. Selected pools were evaluated in terms of Erk2 activation and IM resistance. As it is shown (fig. 4B), cells expressing the hyperactive Mek1 showed a marked resistance to IM. In fact this result demonstrates unequivocally that Erk2 activation is “*per se*” a mechanism of resistance regardless of other molecules/pathways activated/inhibited by Bcr/Abl or any previous exposure to IM.

### Lack of p38MAPK or Erk5 involvement in acquired resistance to IM

**Figure 5:**
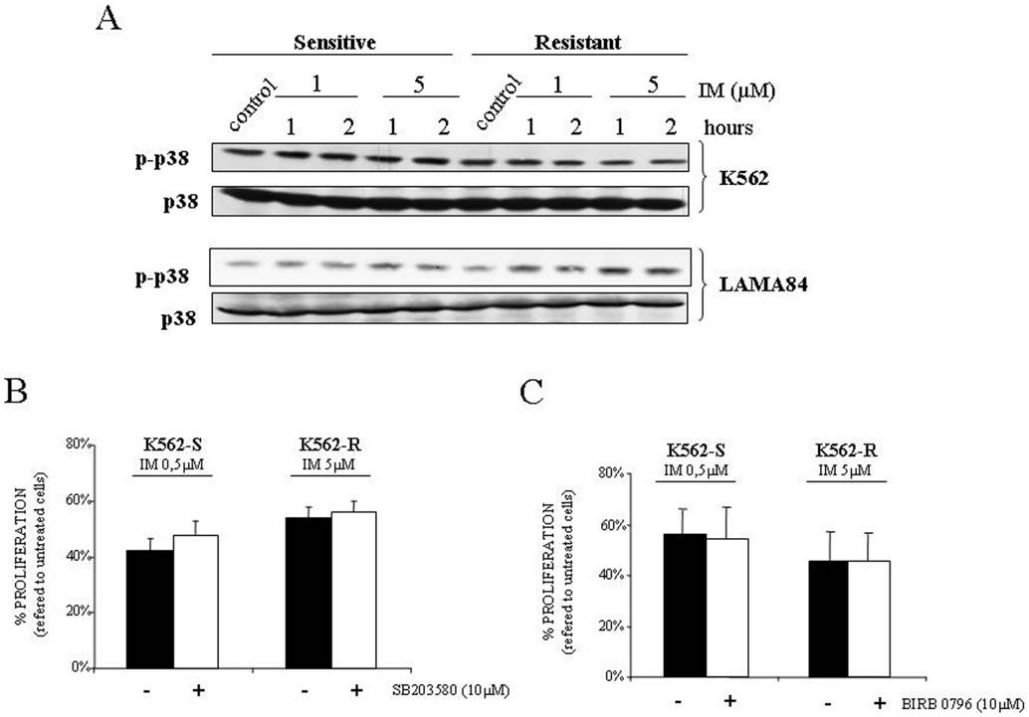
P38MAPK is not implicated in the response to IM. (A) Sensitive and resistant K562 and Lama84 cell lines were treated with indicated concentrations of IM for 1 and 2 hours, lysed and analyzed by immunoblotting against active and total p38MAPK by using 50 µg of total cell lysates. As a loading control membranes were re-probed against tubulin (data not shown). (B) K562-S and K562-R were incubated with the indicated amount of IM in the presence (white bars) or absence (black bars) of SB203580 and then viability was determined 48 hours later. The histogram represents at least 3 independent experiments performed in triplicate cultures. Bars: mean±Standard Deviation. (C) Same as in B but using BIRB0796. Viability was referred to controls treated only with SB203580 or BIRB0796 (in the case of co-incubation with inhibitor) or vehicle (DMSO) in the case of IM alone. Treatment with SB 203580 or BIRB0796 alone induces toxicity lower than 2% in resistant and sensitive cell line.

Two other members of the MAPK superfamily, Erk5 and p38MAPK, have been related to IM resistance [2], [25]. To evaluate the specificity of our observation about the role of ERK2 in IM resistance we decided to study these two MAPKs. First, we studied the p38MAPK signaling pathway and no differences in the activation status were detected in any of the cell lines tested, regardless the presence/absence of IM (fig. 5A). Anyway, to definitively discard the possible role of p38MAPK signaling pathway we used pyridinyl imidazole SB203580, a compound known to inhibit the two main members of this MAPK subfamily p38α and p38β [26]. As shown in figure 5B, no effect was observed due to the presence of the p38MAPK inhibitor. In addition, SB203580 did not modify the response to IM of Lama 84 sensitive and resistant cell lines (Figure S5). Furthermore, to exclude all the p38MAPK family members we used the highly specific inhibitor of all p38MAPKs, BIRB0976 [27] showing the same effect as SB203580 (fig. 5C).

**Figure 6:**
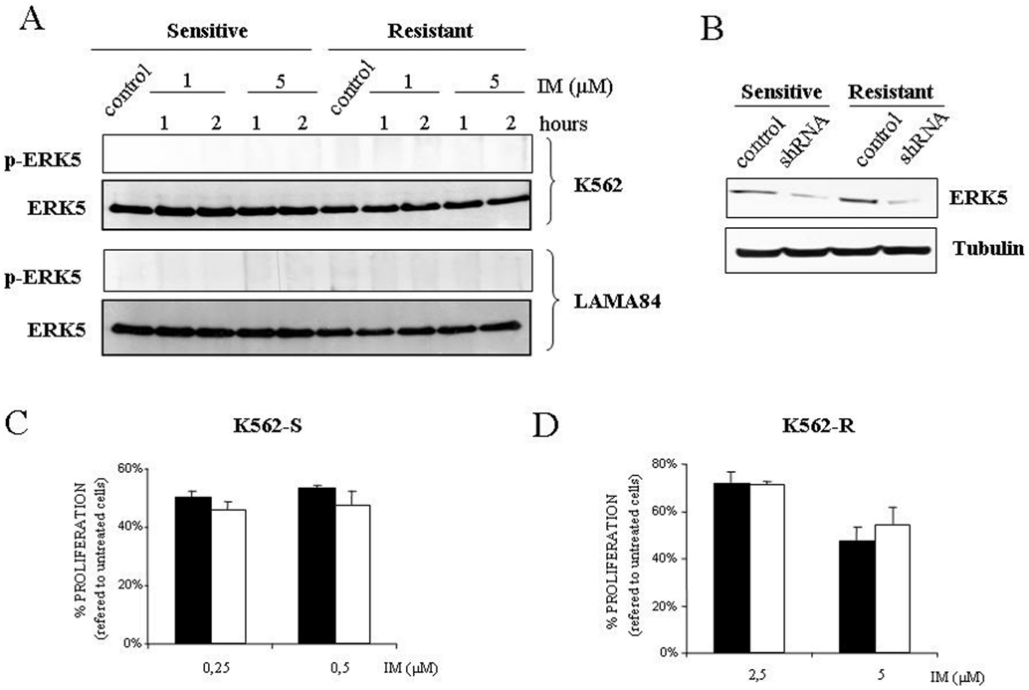
Lack of correlation between Erk5 signaling pathway and resistance to IM. (A) Same as in Fig. 5A but using 120 µg of total cell lysates blotted against indicated antibodies. As a loading control membranes were re-probed against tubulin. (B) Western blot analysis against Erk5 in K562 sensitive and resistant cell line with control plasmid or shRNA against *ERK5*. (C) K562 sensitive cells transfected with shRNA control (black bars) and shRNA against *ERK5* (white bars) were exposed to the indicated concentrations of IM. 48 hours after this treatment viability was determined by MTS assay. The histogram represents at least 3 independent experiments performed in triplicate cultures. Bars: mean±Standard Deviation. (D) Same as in C but in K562 resistant cells.

Regarding to Erk5 no variation in terms of amount/dual phosphorylation was detected in any of cell lines tested regardless the presence/absence of IM (fig. 6A). Considering that no specific inhibitor for this particular MAPK is available, retroviral vectors containing shRNA against *ERK5* were transduced onto resistant and sensitive cell lines (fig. 6B). A marked decrease (>50%) was achieved in Erk5 expression levels, but no significant effect was observed in the response to IM (fig. 6 C and D).

Therefore, the presented results suggest a lack of implication for these two particular MAPKs in the response to IM and support a specific and unique role for Erk2 in our experimental system.

## Discussion

Several conclusions can be obtained from the present study. We found that not all the MAPK family members seem to be critical mediators in the acquired resistance to IM. In this sense, several MAPKs members have been related to IM resistance, but each kinase has been evaluated in a different cell line. Therefore, to avoid any variation due to the cell context, we evaluated these MAPKs in the same experimental system in terms of IM resistance. It is noteworthy that in our experimental model, K562 and Lama84 cell lines, both derived from patients in blast crisis, the resistant variants show a marked increase in Bcr/Abl expression levels, obviously implicated in IM resistance. Interestingly, it has been reported that in K562, the resistant variant did not increase the Bcr/Abl expression levels [13]. However, other report, as well as our data, demonstrate an increase in the Bcr/Abl protein expression [12] in the same experimental model. This apparent discrepancy could be explained, among several possibilities, by the maintenance of cultures for long periods of time in the presence of IM, which probably could select clones with higher levels of Bcr/Abl expression as has been recently demonstrated [28].

Regarding the p38MAPK signaling pathway it has been reported to have a central role in *BCR/ABL* associated transformation [29], [30] as well as in the resistance to other antitumoral agents used in CML [3], [31]. Although it has been considered as a key molecule in the therapeutic effect of IM [25], our data indicate a lack of involvement for p38MAPK in the response to this antitumor agent. Several differences should be considered between our data and those from Parmar and colleagues. First, a different experimental system was used, and second and more important, this previous report studied the role of this MAPK in *de novo* resistance. Interestingly, we did no detect any effect by the inhibition of p38MAPK in K562 parental cells as well as in the Lama84 parental cell line, indicating a marginal role in *de novo* resistance at least in our experimental system. Supporting our data, there are two key biochemical evidences. On the one hand, p38MAPK is activated by Bcr [3], [32], on which the effect of IM is null; and on the other hand, it has been recently reported that c-Abl activates p38MAPK through the stabilization of Mkk6 in a tyrosine kinase independent fashion [20]. Altogether, these data suggest a discrete role for the p38MAPK signaling pathway in the resistance to IM, or at least in the acquired one.

In addition, our results also discard another MAPK superfamily member, Erk5. The stability of this particular MAPK seems to be controlled by c-Abl and Bcr/Abl and also has been postulated to be a critical player in terms of *de novo* resistance [2]. In this regard we have been able to reproduce the control exerted by Bcr/Abl onto Erk5 stability (data not shown), but we failed to demonstrate a role for Erk5 in IM resistance in K562 cells. However, we cannot fully exclude that in certain circumstances, for example increased Erk5 expression, this kinase may contribute to IM resistance as has been demonstrated for MEG01, KG-1 or Ku812 cells [2].

But the connection between MAPKs and CML therapy is not restricted to Erk5 or p38MAPK. A previous report demonstrated a critical role for Erk1/2 signaling pathway in IM response [12]. In this sense the specificity of Erk2 in IM resistance is one of the most important conclusions of the present report. This conclusion is clearly inferred from several experimental data. First, Lama84 and K562 showed a constitutive activation of Erk2 in the resistant cell lines, while Erk1 remained almost undetectable. Furthermore, our data obtained in transient transfection assays demonstrate the specificity of Bcr/Abl for Erk2 activation. In fact this is the first evidence of a specific activation of Erk2 by Bcr/Abl, excluding Erk1, demonstrating a new differential role for these two close MAPKs in CML. This observation, specificity for Erk2 but not for Erk1 -although surprising- is not unique. Probably a differential affinity of Bcr/Abl for the MAPKKs of this signaling pathway could be a plausible explanation. In this regard it has been previously demonstrated how Bcr/Abl activates p38MAPK through both upstream molecules Mkk3 and Mkk6, but c-Abl only affects Mkk6 [3], [32]. This observation could be extremely important, considering that the effect of Bcr/Abl onto Erk2 is only mediated through c-Abl part of the chimeric protein as we have shown. The second experimental evidence came from the specific knock down of *ERK2*, but not of *ERK1*, which increases the IM associated toxicity in resistant cell lines and has no effect in the parental cell lines. And finally, constitutive activation of Erk2 renders resistance in sensitive cell lines, indicating that no other signaling pathway is required for the IM resistance mediated through Erk2. The specific role of Erk2 in IM resistance could be explained, among several possibilities, by selective target for this particular MAPK. For example, it has been reported that BCL_XL_ and BIM -member of the Bcl2 family- are modulated by Erk2 but not by Erk1 [33]. Interestingly, Bim is a proapoptotic molecule [34], critical in IM antileukemic effect [35]. Nonetheless, further studies are necessary to fully understand the exclusive role of Erk2 in IM resistance as well as the universal character of this observation.

It has been reported that CML CD34+ derived cells display basal Erk phosphorylation which is increased in the presence of IM [36], but several differences should be considered between our data and those from Chu et al. First, our data are obtained fundamentally by using cell lines derived from blast crisis, while in the report from Chu and co-workers the conclusions are obtained mainly from patients in chronic phase. Second, different time of exposure to IM has been used. In this sense there are reports that demonstrate activation of Erk1/2 by IM after 24 hours of treatment in K562 cells [12], while also it has been reported an inhibitory effect in similar conditions [37]. Therefore, to avoid this discrepancy as wells as to find specific biochemical events unrelated to toxicity we decided to use short time incubations, that could explain the lack of effect in Erk2 phosphorylation observed. Finally, the presence of specific growth factor for CD34+ cells can modulate the activation of Erk1/2 in response to IM. In fact, it has been recently report by the same group [38], how the presence/absence of growth factors differentially modulate several signalling pathways, including MAPK, in response to Nilotinib, which interestingly decrease the levels of phosphor-Erk2 in K562 cells [39].

From the therapeutic point of view, our report supports a role for Erk2 in the acquired and in *de novo* resistance to IM. For example, regarding *de novo* resistance, our experimental approach using an active H-Ras, K-Ras and constitutively active Mek1 demonstrate the critical role of Erk2. In fact, *Ras* activating mutation has been related with disease progression, for H and N-Ras protoncogenes [40], and more recently it has been reported a critical role for *K-Ras* mutations in IM resistance [19]. However, *Ras* genes could explain the resistance in few patients due to the low incidence of *Ras* mutation detected in CML [41]. But the vast majority of IM therapy failure -excluding those associated to mutation in the Bcr/Abl oncoprotein [42]- occur in advanced stage of the disease in which Bcr/Abl overexpression is a hallmark of the disease's progression [16], [43] and could account for more than 50% of the IM resistant cases [44]. Our data using cell lines and primary patient samples support the correlation between Erk2 activation and Bcr/Abl levels in the generation of an IM resistant phenotype, suggesting that the use of Erk2 signaling pathway inhibitors -that target the Ras/Raf/ERK route- could represent a complementary therapy in those patients with advanced stages of CML, especially in those who are refractory to IM based therapy [45]. Furthermore, Erk2 could also be involved in the resistance to IM related to sphingosine kinase-1 (SphK1) as has been recently proposed [46]. In addition, our results could probably apply to the new generation of specific inhibitors for c-Abl, such as Dasatinib or Nilotinib [39]. Finally, the activation of Erk2 could be a critical issue in the pathogenesis of the disease, considering the importance of Ras signaling pathway in *BCR/ABL* dependent transformation [47], [48]. In fact it has been reported how in response to Ras, Erk2 has a positive role in cell proliferation while Erk1 antagonize Erk2 activity [49]. Furthermore, a critical molecule in cellular transformation as p21WAF has been proposed to be a selective target of Erk2 but not for ERK1 [50]. Furthermore, regarding the progression of the disease it has been demonstrated a critical role for Erk1/2 signaling pathway in the blast crisis of CML through the suppression of the normal myeloid differentiation to neutrophil in a Bcr/Abl dose dependent fashion [51]. Therefore, the selective activation of Erk2 by Bcr/Abl can render specific biological properties, ranging from transformation up to IM resistance, due to the effect on exclusive targets for this MAPK.

In summary, this work demonstrates a critical role for Erk2 in the acquired and in *de novo* resistance to IM, while at least two other members of the MAPK superfamily, Erk5 and p38MAPK, seem to have a marginal role, if any. Our data support the use of new therapeutic approaches based on the inhibition of Erk2 signaling pathway that will improve the current therapy of CML, even in the advanced stage of the disease were overexpression of Bcr/Abl is a common feature.

## Supporting Information

Figure S1 Role of ERK1/2 localization
(0.13 MB TIF)

Figure S2 Effect of PD98059 on Lama84
(0.07 MB TIF)

Figure S3 Characteristics of patients
(0.07 MB TIF)

Figure S4 K-ras mutations and ERK1/2 activation
(0.11 MB TIF)

Figure S5 Effect of SB203580 on Lama 84
(0.07 MB TIF)
