# Supplementary figures and images for: ERK2, but Not ERK1, Mediates Acquired and “De novo” Resistance to Imatinib Mesylate: Implication for CML Therapy
Source: PLoS One. 2009 Jul 1;4(7):e6124. doi: 10.1371/journal.pone.0006124 (PMC2699476; doi:10.1371/journal.pone.0006124)

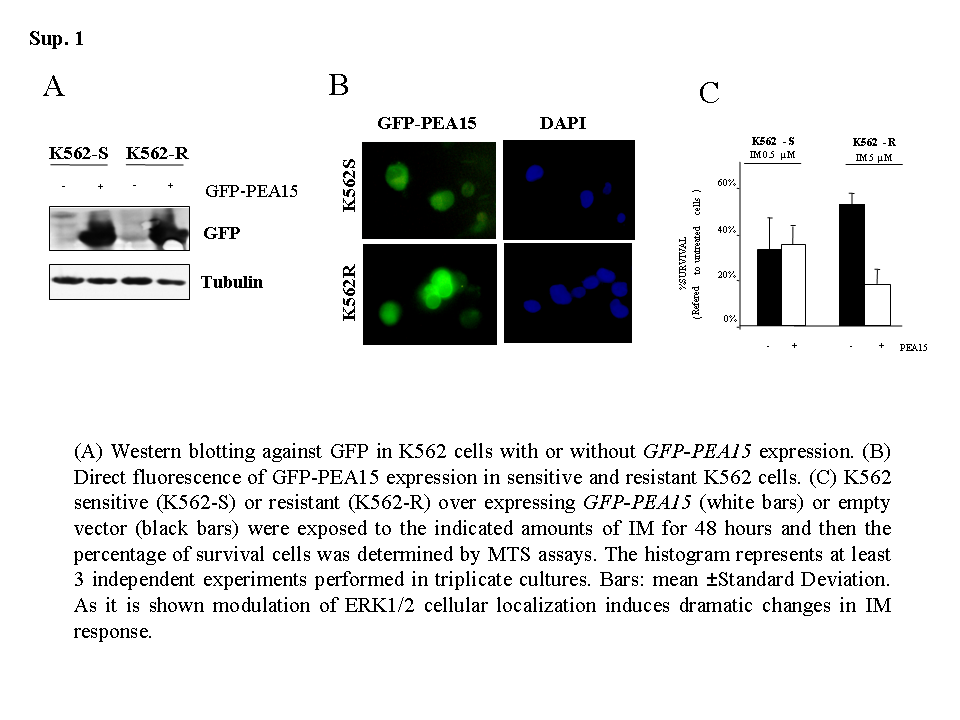

Supplement: Figure S1 — Role of ERK1/2 localization (0.13 MB TIF) [file pone.0006124.s001.tif]

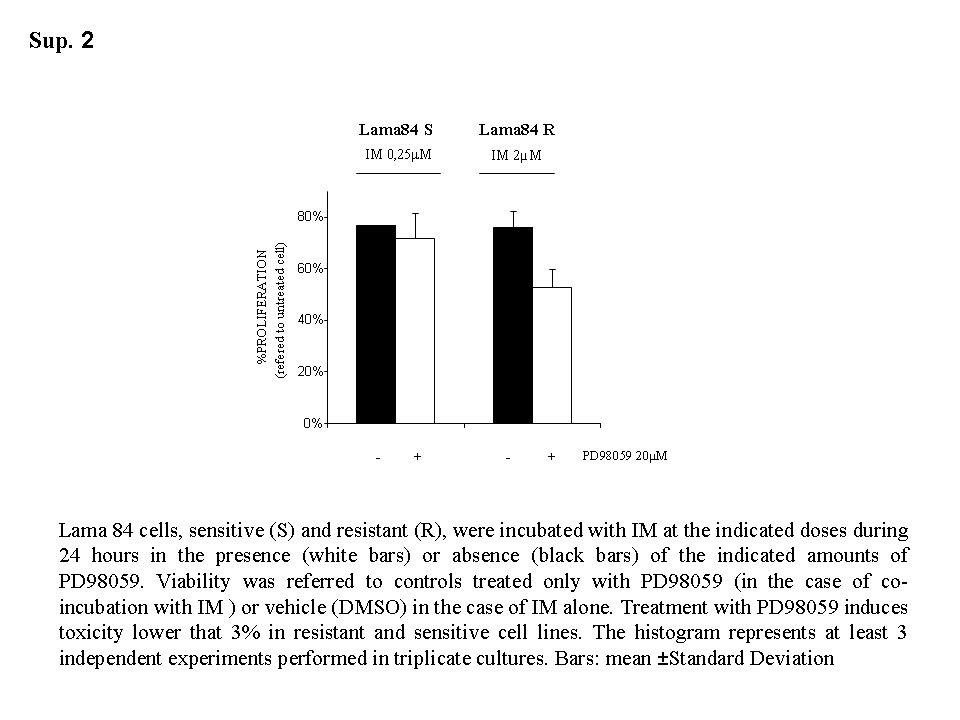

Supplement: Figure S2 — Effect of PD98059 on Lama84 (0.07 MB TIF) [file pone.0006124.s002.tif]

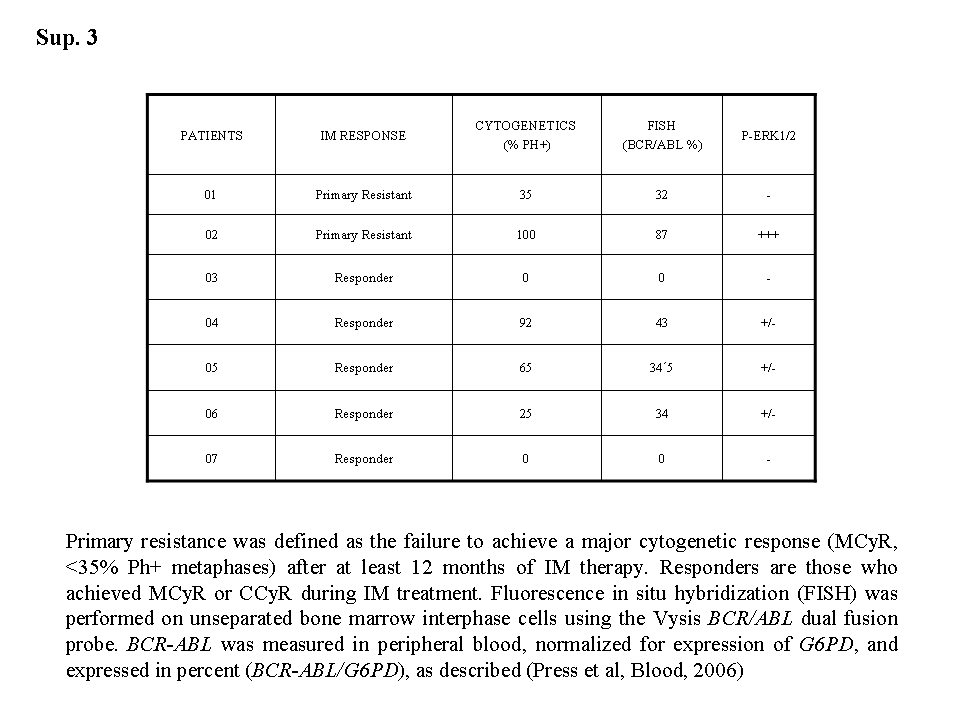

Supplement: Figure S3 — Characteristics of patients (0.07 MB TIF) [file pone.0006124.s003.tif]

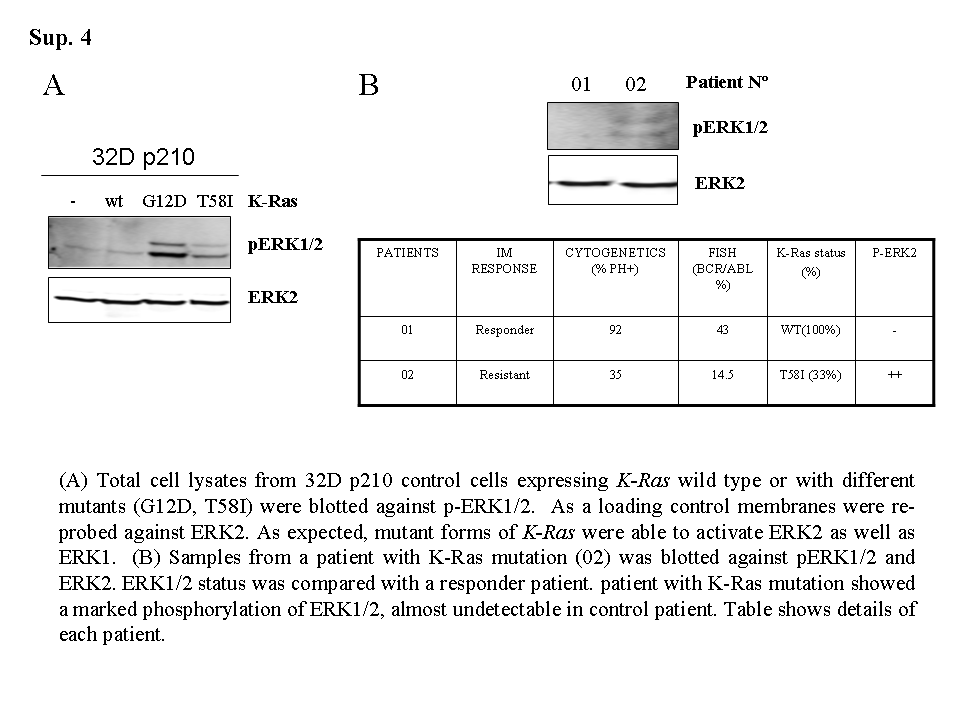

Supplement: Figure S4 — K-ras mutations and ERK1/2 activation (0.11 MB TIF) [file pone.0006124.s004.tif]

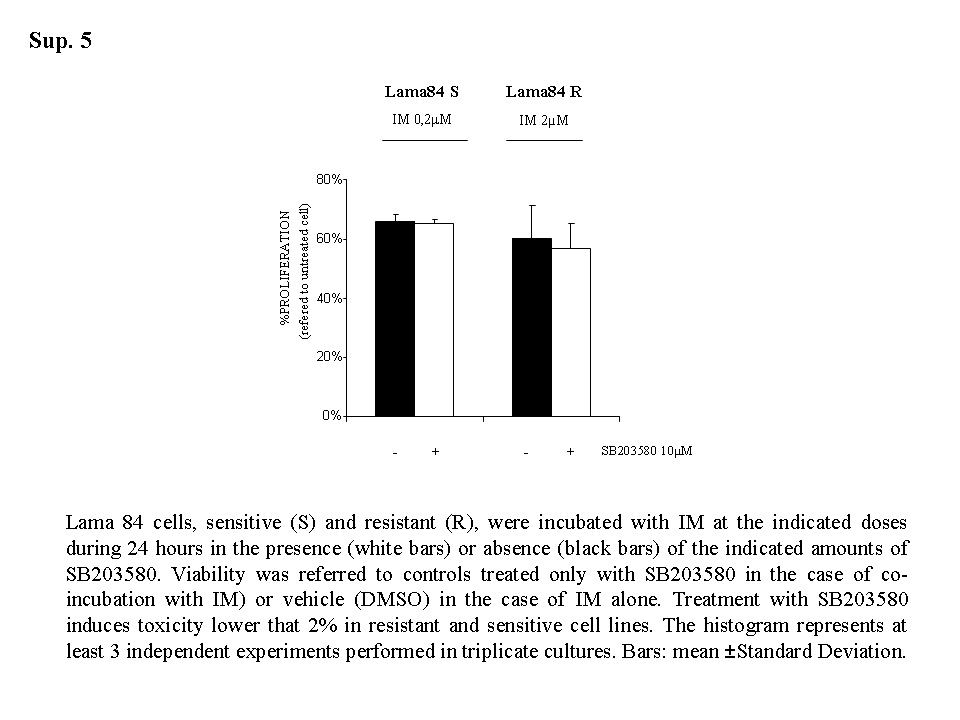

Supplement: Figure S5 — Effect of SB203580 on Lama 84 (0.07 MB TIF) [file pone.0006124.s005.tif]
